# Effects of Hydrogen Gas Inhalation on Community-Dwelling Adults of Various Ages: A Single-Arm, Open-Label, Prospective Clinical Trial

**DOI:** 10.3390/antiox12061241

**Published:** 2023-06-08

**Authors:** Md. Habibur Rahman, Johny Bajgai, Subham Sharma, Eun-Sook Jeong, Seong Hoon Goh, Yeon-Gyu Jang, Cheol-Su Kim, Kyu-Jae Lee

**Affiliations:** 1 Department of Convergence Medicine, Wonju College of Medicine, Yonsei University, Wonju 26426, Gangwon-do, Republic of Koreacs-kim@yonsei.ac.kr (C.-S.K.); 2 Department of Neurosurgery, Wonju Severance Christian Hospital, Yonsei University Wonju College of Medicine, Wonju 26426, Gangwon-do, Republic of Korea

**Keywords:** Alzheimer’s disease, H_2_ gas inhalation, reactive oxygen species, cognitive impairment, dementia biomarkers

## Abstract

Molecular hydrogen (H_2_) is a versatile therapeutic agent. H_2_ gas inhalation is reportedly safe and has a positive impact on a range of illnesses, including Alzheimer’s disease (AD). Herein, we investigated the effects of 4 weeks of H_2_ gas inhalation on community-dwelling adults of various ages. Fifty-four participants, including those who dropped out (5%), were screened and enrolled. The selected participants were treated as a single group without randomization. We evaluated the association between total and differential white blood cell (WBC) counts and AD risk at individual levels after 4 weeks of H_2_ gas inhalation treatment. The total and differential WBC counts were not adversely affected after H_2_ gas inhalation, indicating that it was safe and well tolerated. Investigation of oxidative stress markers such as reactive oxygen species and nitric oxide showed that their levels decreased post-treatment. Furthermore, evaluation of dementia-related biomarkers, such as beta-site APP cleaving enzyme 1 (BACE-1), amyloid beta (Aβ), brain-derived neurotrophic factor (BDNF), vascular endothelial growth factor A (VEGF-A), T-tau, monocyte chemotactic protein-1 (MCP-1), and inflammatory cytokines (interleukin-6), showed that their cognitive condition significantly improved after treatment, in most cases. Collectively, our results indicate that H_2_ gas inhalation may be a good candidate for improving AD with cognitive dysfunction in community-dwelling adults of different ages.

## 1. Introduction

A crucial component of clinical trial inclusion criteria is the use of biomarkers that reliably identify the pathology of Alzheimer’s disease (AD) over the course of an individual’s lifetime. The prevalence of AD is 3% at the age of 65 years, which doubles every 5–6 years [1]. By 2025, South Korea is expected to have a super-aged society, only 8 years after becoming an elderly society. By the year 2045, the proportion of elderly persons is anticipated to increase by 37% [2]. Globally, approximately 50 million people suffer from dementia, with nearly 10 million new cases diagnosed every year [1]. The prevalence of dementia among individuals over 65 years of age in Korea was predicted to be 10.25% in 2020, or approximately 830,000 individuals. However, it is predicted to increase by about 15.91% by 2050, with an additional 3.02 million dementia sufferers [2]. Thus, effective therapeutic and preventive strategies are required to overcome the challenges of this global issue. The most common form of dementia-related neuropathological brain changes can reportedly begin as early as 20 years before symptoms appear; these can be further evaluated with potential biomarkers to determine treatment measures [3]. Normal metabolism in biological systems involves the production of reactive oxygen species (ROS). Free radicals are highly reactive entities that can initiate a chain of reactions that damage the cell. Nevertheless, even under ideal circumstances, cells biologically create free radicals, which are typically neutralized by endogenous enzymatic cellular antioxidant processes [4]. In patients with AD, oxidative stress and cellular damage brought on by protein, lipid, and DNA oxidation appear to precede the development of cognitive deterioration [5]. Therefore, it is important to identify such patients in the community using thorough evaluation that includes biomarker testing, quick cognitive assessments, and confirmatory neurocognitive testing. Because blood-based biomarkers are less invasive, low-risk, and aim for an overall accuracy comparable to that of the more established cerebrospinal fluid (CSF) biomarkers, those directly linked to the pathogenesis of AD have recently become the focus of intense research. Because it causes oxidative stress in the brain, amyloid beta (Aβ), majorly contributes to neurodegeneration in patients with AD [6,7]. The abnormal production and aggregation of Aβ isoforms are pathophysiological hallmarks that start decades before symptoms appear, causing synaptic loss, neuronal death, and clinical dementia. Aβ is the most studied blood biomarker (either plasma or serum). Thus, monitoring amyloid processes in asymptomatic individuals may help identify those who develop AD at the prodromal stage [8]. In addition, other potential biomarkers for the early detection of AD pathology have been found, such as plasma proteins, lipids, Aβ-40, Aβ-42, and their ratio, tau, and beta-secretase 1 (BACE-1) [9,10]. Neurotrophins, such as the brain-derived neurotrophic factor (BDNF), may also be involved in AD pathogenesis [11]. Additionally, reactive astrocytes are linked to the active production of several pro-inflammatory and toxic mediators in the brain of patients with AD, which can result in synaptic dysfunction [12,13]. Interleukin-6 (IL-6) and other inflammatory molecules are also linked to AD [14]. Pathological alterations in AD are linked to the upregulation of several chemokines, including monocyte chemotactic protein-1 (MCP-1) [15]. Age has been linked to higher MCP-1 levels in the Japanese community, which is likely influenced by the aging population [15]. Vascular endothelial growth factors (VEGFs) play a role in the development and maintenance of both vascular and neural cells [16]. Elevated blood–brain barrier (BBB) permeability, pericyte loss, and severe tangle pathology have been linked to increased VEGF levels in the brain of patients with AD [17,18].

Numerous approaches have been recently considered to address age-related memory impairment in clinical settings. One such approach is treatment with molecular hydrogen (H_2_). It is a novel natural antioxidant with a low propensity to react with most biomolecules and excellent therapeutic properties [19]. H_2_ selectively neutralizes free radicals, such as hydroxyl (•OH) and peroxynitrite (ONOO^−^) [19]. A recent study found that administering H_2_ to healthy seniors positively impacts several age-related traits, such as extended mean telomere length and a tendency to increase DNA methylation [20]. The consumption of 1000 mL/d of H_2_-water for 48 weeks reportedly reduces PD severity [21]. Because inhaling 1–4% H_2_ has demonstrated great efficacy in medical applications, the use of H_2_ at such low concentrations has been deemed feasible and safe [22]. Additionally, Hong and colleagues reported that the administration of H_2_ water alleviates disease severity, particularly in the mood and cognitive domains [23]. Another study reported that the administration of H_2_ inhalation improves cognitive function in older women, suggesting that H_2_ is a beneficial agent for patients with age-related cognitive deficits [24]. Despite increasing evidence indicating the beneficial effects of H_2_, studies to evaluate the effectiveness of H_2_ as a blood-based biomarker in an aged healthy population are limited. Thus, we aimed to investigate the effects of 4 weeks of H_2_ inhalation on cognitive performance and blood-based AD biomarkers in community-dwelling adults of various ages.

## 2. Materials and Methods

### 2.1. Ethics Approval

Ethical guidelines for medical research using human participants were implemented during the study. Clinical research participants were recruited through a recruitment notice from researchers. The study was approved by Yonsei University Wonju Severance Christian Hospital’s Research Review Board (No: CR323002) and registered with Clinical Trials.gov under the identifier NCT05891938. Patients were included only if a signed consent was obtained. Participants were free to withdraw from the experiment at any point. The questionnaires in the consent form were not critical of the participants. All the findings of this investigation were kept private and confidential.

**Figure 1:**
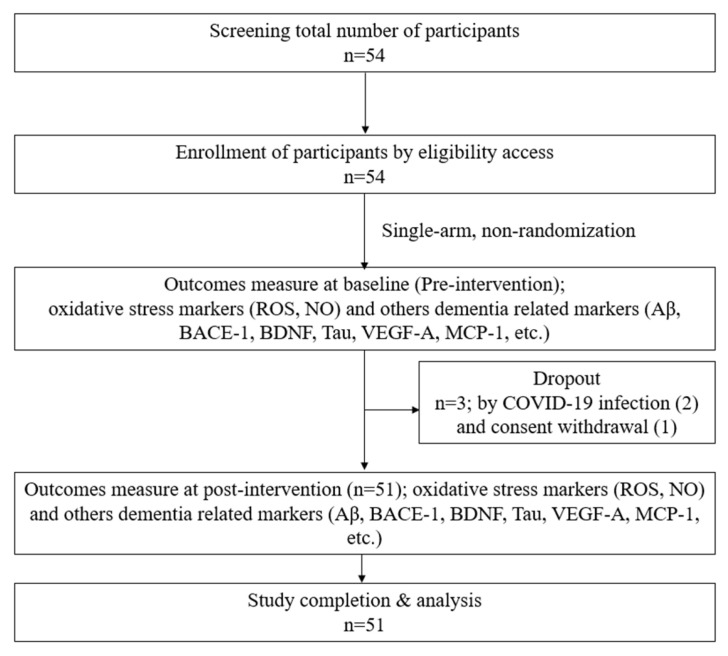
Study design.

Following approval from Yonsei University Wonju Severance Christian Hospital’s IRB, subjects signed a permission form. Male and female participants who met the predetermined criteria were selected. The willing participants were assigned screening numbers. The physical examination data (height and weight), demographic data (sex and age), medical/medication history, and vital signs (temperature, blood pressure, and pulse) of each patient were recorded. Finally, 51 participants who met the inclusion criteria were selected for this study design, as shown in Figure 1. The basic characteristics, vital signs, accompanying symptoms, psychological problems, and recent lifestyle changes of the included participants were recorded. H_2_ gas 2–3% was produced from an H_2_-generating device designed and provided by the company (GOOTZ Co., Ltd., Yangju-si, Gyeonggi-do, Republic of Korea). Therefore, 2% hydrogen + 98% filtering air inhalation (30 min/day) with a nasal cannula was provided for 4 weeks. The inclusion and exclusion criteria are listed below in Table 1. In addition, gender, education, systolic blood pressure, and diastolic blood pressure were recorded in this study to obtain baseline data, as shown in Table 2.

### 2.2. Differential White Blood Cell Count Analysis

Retro-orbital plexus blood was collected and placed in tubes coated with ethylenediaminetetraacetic acid (EDTA). The total and differential white blood cell (WBC) counts, including those of lymphocytes, monocytes, neutrophils, eosinophils, and basophils, were measured using an automatic blood analyzer (HEMAVET HV950 FS; Drew Scientific Inc., Dallas, TX, USA).

### 2.3. Total ROS Estimation

Using the oxidation of 2–4-dichlorodihydrofluorescein diacetate (DCFH-DA) (Abcam, Cambridge, MA, USA), the total ROS production in serum was assessed. A total of 50 µL of serum samples was placed in a 96-well plate. After adding 100 µL of 10µM DCFH-DA to the wells, the plate was incubated in the dark for 30 min. The plate was then read at 488 nm excitation/525 nm emission using a DTX-880 multimode microplate reader (Beckman Counter Inc., Fullerton, CA, USA).

### 2.4. Nitrous Oxide Level Estimation

Griess reagent (Promega Corp., Madison, WI, USA) was used to measure NO generation in blood; the test was performed according to the manufacturer’s instructions. Briefly, standards were prepared, and nitrite measurement was performed by adding 50 µL of serum samples into the wells. In each well, 50 µL of sulfanilamide solution was added and incubated for 10 min in the dark at room temperature. Then, in each well, 50 µL of N-(1-Naphthyl) ethylenediamine (NED) solution was added and incubated for 15 min in the dark at room temperature. A SpectraMax^®^ ABS Plus (Molecular Devices, San Jose, CA, USA) was used to measure the OD at 520 nm.

### 2.5. BDNF Level Measurement

Serum levels of total BDNF were measured using a human BDNF ELISA Kit (catalog number: EH42RB). The test was performed in accordance with the manufacturer’s instructions. Serum samples (20 µL) were added to constructed standards and incubated for 2.5 h at room temperature. The samples were washed three times. Subsequently, 100 µL of biotin conjugate was added and incubated for 1 h. The samples were washed three times and 100 µL of Streptavidin-HRP solution was added in each well. These were incubated again for 45 min. Thereafter, 100 µL of this solution was added to the TMB substrate in each well and incubated for 30 min at room temperature. SpectraMax^®^ ABS Plus (Molecular Devices, San Jose, CA, USA) was used to measure the OD at 450 nm.

### 2.6. BACE-1 Level Measurement

Serum levels of total BACE-1 were measured using a human BACE-1 ELISA Kit (catalog number: ab267637). The test was performed in accordance with the manufacturer’s instructions, such as that carried out for BACE-1 level estimation. Serum samples (20 µL) were added to constructed standards and incubated for 2.5 h at room temperature. The samples were washed three times. Subsequently, 100 µL of biotin conjugate was added and incubated for 1 h. The samples were washed three times and 100 µL of Streptavidin-HRP solution was added in each well. These were incubated again for 45 min. Thereafter, 100 µL of this solution was added to the TMB substrate in each well and incubated for 30 min at room temperature. SpectraMax^®^ ABS Plus (Molecular Devices, San Jose, CA, USA) was used to measure the OD at 450 nm.

### 2.7. Detection of Inflammatory Cytokines, Aβ, and Tau Protein by Multiplex Assay

The previously described approach was utilized to test the blood levels of inflammatory cytokines, including IL-6, MCP-1, Aβ, t-tau, p-tau, and VEGF-A using a Bead Array Suspension Multiplex Kit (Bio-Rad, San Diego, CA, USA). Each standard concentration was resuspended in standard diluents to create serial dilutions of the standard. The bead combination was mixed with the typical serum sample. After being washed, the plate was incubated for a further 18 h at 4 °C. The plate was then incubated for an hour at room temperature after the addition of the detecting antibody. The plate was then coated with streptavidin–phycoerythrin solution and left to sit at room temperature for 30 min. The plate was then examined using the (Millipore Corporation, Billerica, MA, USA) after the washing process; an assay buffer was then applied.

### 2.8. Data Management and Statistical Analysis

Statistical analysis was carried out using Graph Pad Prism (version 8.0; GraphPad Software, La Jolla, CA, USA) using unpaired *t*-tests. Data are expressed as mean ± standard deviation (SD). Differences were considered statistically significant at *p* < 0.05.

## 3. Results

### 3.1. Effects of H_2_ Gas Inhalation on Total and Differential WBC Counts in Community-Dwelling Adults of Different Ages

**Figure 2:**
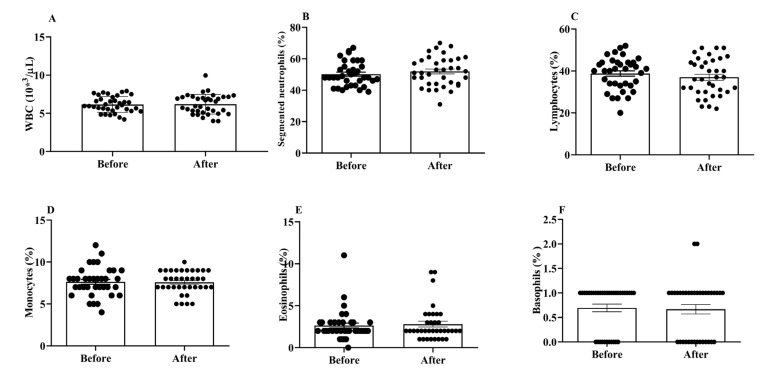
Effects of H_2_ gas inhalation on total and differential WBC counts, as well as the variations in these counts among residents of different ages in the community. (**A**) White blood cell (WBC); (**B**) Segmented neutrophils; (**C**) Lymphocytes; (**D**) Monocytes; (**E**) Eosinophils; (**F**) Basophils. Data are presented as mean ± SD.

WBCs are important for both the innate and adaptive immune responses in the body. We did not find any significant changes in the total and differential WBC counts between the post- and pre-treatment values (Figure 2A–F).

### 3.2. Effects of H_2_ Gas Inhalation on ROS and NO in Community-Dwelling Adults of Different Ages

**Figure 3:**
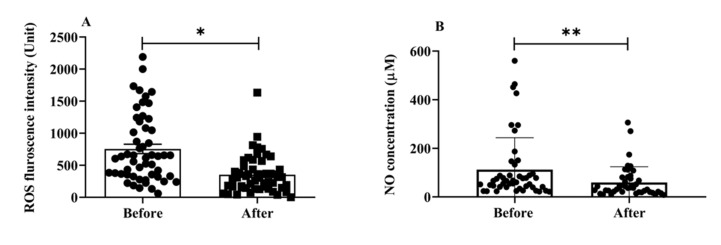
Effects of consuming H_2_ gas on serum levels of ROS and NO in community-dwelling persons of various ages, both before and after therapy. (**A**) ROS fluorescence intensity; (**B**) NO concentration (μM). Data are presented as mean ± SD. ** *p* < 0.01, * *p* < 0.05.

Oxidative stress is involved in the progression of aging and AD [25]; it is one of the main mechanisms underlying cognitive aging and neurodegenerative diseases. We estimated the total ROS and NO serum levels to examine the impact of H_2_ gas inhalation on community-dwelling adults of various ages. The serum levels of total intracellular ROS (*p* < 0.05) (Figure 3A) and NO (*p* < 0.01) (Figure 3B) significantly decreased after treatment. Both OS indicators, ROS and NO, indicate that H_2_ gas is useful in treating cognitive impairment community-dwelling adults of various ages after 4 weeks of treatment.

### 3.3. Effects of H_2_ Gas Inhalation on Serum BACE-1 Levels in Community-Dwelling Adults of Various Ages

**Figure 4:**
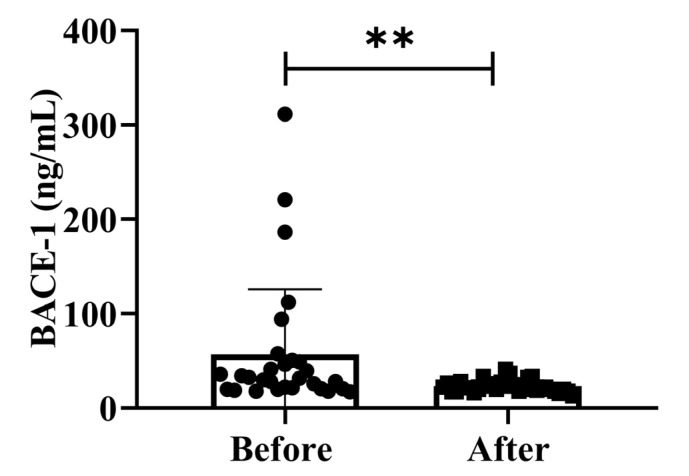
Effects of H_2_ gas inhalation on serum BACE-1 levels before and after treatment. Data are presented as mean ± SD. ** *p* < 0.01.

BACE-1 levels activity is isolated from post-mortem human brains since AD neuropathology primarily develops in the cortex and hippocampus. We assessed the effect of possible confounding variables on the relationship between BACE-1 serum activity and cognitive diagnosis on community-dwelling adults of various ages. The total intracellular BACE-1 levels (*p* < 0.01) significantly decreased after treatment (Figure 4). Additionally, BACE-1 levels are shown in different ages (50–59 years, 60–69 years, and 70–79 years) separately in Supplementary Figure S1. Our findings demonstrated that the dementia marker BACE-1 was effective in reducing dementia on community-dwelling individuals of various ages after 4 weeks of treatment; (50–59 years; * *p* < 0.05), (60–69 years; * *p* < 0.05), and (70–79 years; * *p* < 0.05) are shown in Figure S1.

### 3.4. Effects of H_2_ Gas Inhalation on the Serum BDNF Levels in Community-Dwelling Adults of Various Ages

**Figure 5:**
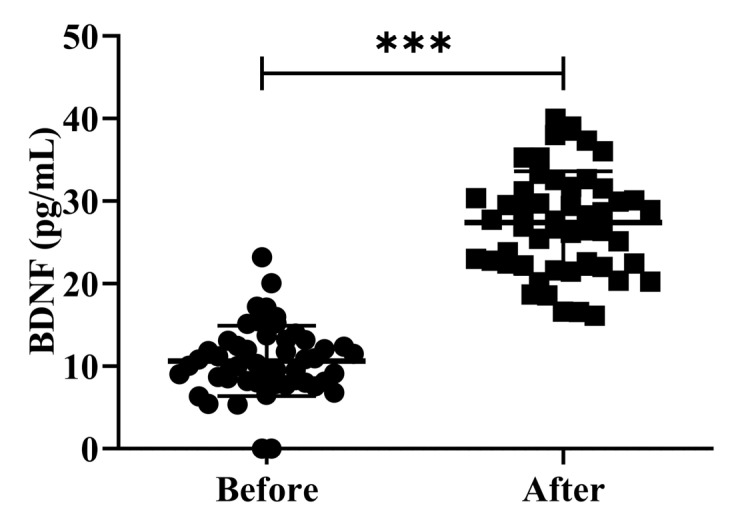
Impact of H_2_ gas inhalation on serum BDNF levels before and after treatment. Data are presented as mean ± SD. *** *p* < 0.001.

The investigation of peripheral BDNF levels in clinical research involving patients is controversial. While several studies have shown that patients with AD have greater peripheral BDNF levels than healthy controls, other studies have found the opposite. Earlier studies examining the levels of serum BDNF in patients with AD and MCI have reported conflicting results [26]. In the present study, we investigated the effects of H_2_ gas inhalation on serum BDNF levels on community-dwelling adults of various ages. The serum BDNF levels were significantly increased (*p* < 0.001) after treatment on community-dwelling adults of various ages as compared to before treatment; the results are shown in Figure 5. Our results confirmed that the dementia marker BDNF was effective in reducing cognitive impairment on community-dwelling individuals of various ages after 4 weeks of treatment; (50–59 years; *** *p* < 0.001), (60–69 years; *** *p* < 0.001), and (70–79 years; *** *p* < 0.001) are shown Figure S2.

### 3.5. Effects of H_2_ Gas Inhalation on Serum Levels of Aβ-40, Aβ-42, t-Tau and p-Tau in Community-Dwelling Adults of Various Ages

**Figure 6:**
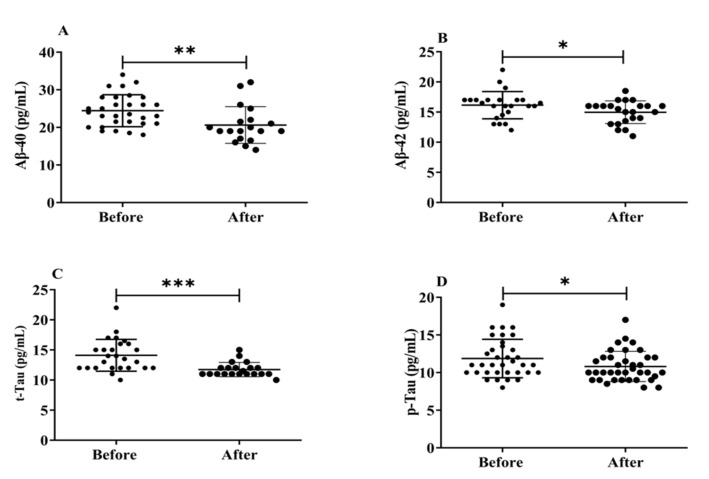
Effects of H_2_ gas inhalation on serum levels of Aβ-40, Aβ-42, t-Tau, and p-Tau before and after treatment. (**A**) Aβ-40 (pg/mL); (**B**) Aβ-42 (pg/mL); (**C**) t-Tau (pg/mL); (**D**) p-Tau (pg/mL). Data are presented as mean ± SD. *** *p* < 0.001, ** *p* < 0.01, * *p* < 0.05.

Neurofibrillary amyloid plaques, abnormal protein deposits made up of Aβ peptides (Aβ1-40 and Aβ1-42), and build-up of intracellular insoluble hyperphosphorylated tau proteins (p-Tau) that are the hallmarks of AD brain pathology. Herein, we evaluated how exposure to H_2_ gas affects the serum levels of Aβ peptides (Aβ1-40, Aβ1-42), t-tau, and p-Tau on community-dwelling adults of various ages. There were significant differences found in all biomarkers, such as Aβ1-40 (*p* < 0.01), Aβ1-42 (*p* < 0.05), t-tau (*p* < 0.001), and p-Tau (*p* < 0.05), after treatment compared to before treatment (Figure 6). Our results demonstrated that the dementia marker, (Aβ1-40 and Aβ1-42), levels reduced significantly on community-dwelling individuals of various ages after 4 weeks of treatment; (Aβ1-40 and Aβ1-42; 50–59 years; * *p* < 0.05, 60–69 years; * *p* < 0.05; 70–79 years; * *p* < 0.05) are shown in Figure S3; similarly, t-tau and p-tau levels reduced dramatically in different ages within 4 weeks of treatment; (60–69 years; ** *p* < 0.01; 70–79 years; * *p* < 0.05) are shown Figure S4.

### 3.6. Effects of H_2_ Gas Inhalation on Serum Levels of MCP-1, IL-6, and VEGF-A in Community-Dwelling Adults of Various Ages

**Figure 7:**
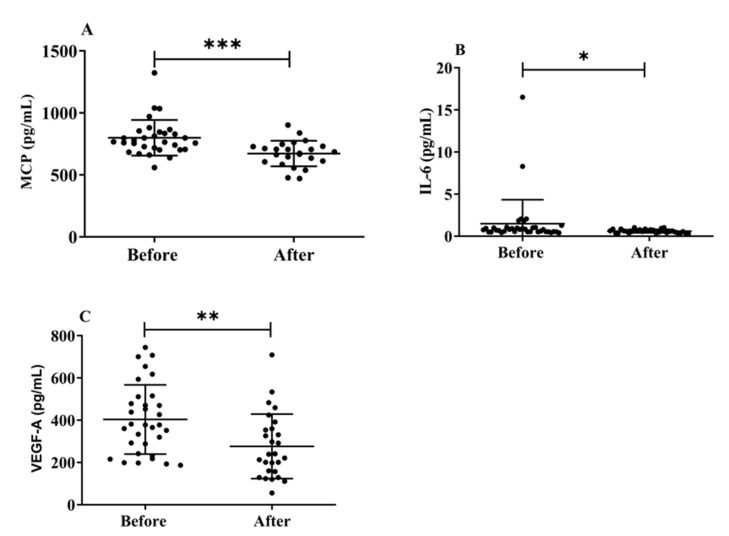
Effects of H_2_ gas inhalation on the levels of IL-6, MCP-1, and VEGF-A in the serum before and after treatment in community-dwelling people of various ages. (**A**) Monocyte chemotactic protein-1 (MCP), (pg/mL); (**B**) IL-6 (pg/mL); (**C**) VEGF-A (pg/mL). Data are presented as mean ± SD. *** *p* < 0.001, ** *p* < 0.01, * *p* < 0.05.

Herein, we investigated the effects of H_2_ gas inhalation on the serum levels of IL-6, MCP-1, and VEGF-A on community-dwelling adults of various ages. The MCP-1 (*p* < 0.001) (Figure 7A), IL-6 (*p* < 0.05) (Figure 7B), and VEGF-A (Figure 7C) levels significantly decreased after treatment on community-dwelling adults of various ages compared to before treatment. Our results indicated that the dementia marker MCP-1 was effective in reducing cognitive impairment on community-dwelling individuals of various ages after 4 weeks of treatment; (50–59 years; * *p* < 0.05), (60–69 years; *** *p* < 0.001), and (70–79 years; ** *p* < 0.01) are shown Figure S5. Furthermore, VEGF-A levels also significantly decreased after treatment on community-dwelling adults of various ages compared to before treatment; (50–59 years; * *p* < 0.05), (60–69 years; * *p* < 0.05), and (70–79 years; * *p* < 0.05) are shown in Figure S5.

## 4. Discussion

This study was designed to investigate the effectiveness of H_2_ gas inhalation in adults of different ages. The results of the present clinical trial support the effectiveness of H_2_ gas inhalation in improving cognitive impairment and related symptoms in patients with dementia. Moreover, no adverse effects were recorded after four weeks of treatment with H_2_ gas inhalation, and therefore, it can be considered a safe and well-tolerated healthy supplement for the treatment of AD in community-dwelling adults of various ages. Currently, no blood-based biomarkers can detect AD in its preclinical state. Aβ plaque and neurofibrillary tangles develop as a result of inflammation and immunological dysfunction. By stimulating microglial cells, the immune response initially lowers the plaque burden against neurodegeneration [27]. To stop the buildup of plaque, more inflammatory cytokines are released, and macrophage numbers increase [28]. During this stage, there is an increase in the number of peripheral blood cells, including lymphocytes, neutrophils, monocytes, platelets, and lymphocyte subsets, which are involved in inflammation and the immune response [29]. Numerous investigations have demonstrated that patients with AD and MCI had drastically increased peripheral neutrophil counts or decreased peripheral lymphocyte numbers [29]. Our results demonstrated that H_2_ gas inhalation treatment did not cause any adverse effects, indicating that it was safe. To date, no reliable conclusion has been made regarding the alterations in peripheral blood cell profiles. Sensitivity and other technological constraints have slowed the development of blood-based biomarkers. Age-related diseases, their developmental stages, and cell signaling pathways can all be linked to ROS generation [30,31]. Neuronal cells are more susceptible to oxidative stress than other normal body tissues owing to their high oxygen consumption, high lipid content, and lack of antioxidant enzymes [32]. One of the major factors causing AD pathogenesis is the brain’s vulnerability to ROS. Oxidative stress irreversibly damages cellular biomolecules and disrupts neuronal activity [32]. Our results suggest that H_2_ gas inhalation improves cognitive function in patients with dementia by decreasing ROS levels. Due to its crucial physiological role, NO is involved in a variety of neurological disorders, including AD and other neurodegenerative dementias, according to a growing body of research [32]. According to numerous studies, the number of neurons in the hippocampus and BDNF concentrations in the cranial cortex were both found to increase following ischemic brain injury. An asymptomatic inflammatory process is the best description of the chain of events caused by chronic diseases and aging [33]. These findings highlight the neuroprotective function of BDNF in ischemic brain damage. Postmortem examination of the AD brain revealed a higher BDNF concentration which is shown in Figure 4 and Figure S2. This study also advances the theory that a compensatory mechanism boosts the concentration of BDNF [34]. BACE-1 is a novel type 1 transmembrane aspartic acid protease that shares 501 amino acids with pepsin and retroviral aspartic proteases [35]. In the amyloidogenic pathway, BACE1 (β-secretase) breaks down the amyloid precursor protein (APP) to produce Aβ. As a result, the therapeutic inhibition of β-secretase would result in a reduction in the synthesis of all Aβ forms, including the pathogenic Aβ-42 [36]. Therefore, it is crucial to understand the biochemistry of BACE-1 to identify possible therapeutic targets in the etiology of AD. Our study demonstrated that after four weeks, H_2_ gas inhalation treatment significantly decreased BACE-1 levels compared to the pre-treatment group. Our results indicate that H_2_ gas inhalation may be a good candidate for improving cognitive impairment in patients with dementia, as demonstrated in Figure 3 and Figure S1.

The development of tau neurofibrillary tangles and amyloid plaques inside brain tissues are both associated with AD. Brain damage from neurotoxic peptides, such as soluble or fibrillar Aβ, may be more likely to occur as a result of age-related accumulation of oxidative stress metabolites. Additionally, the buildup of Aβ can lead to an increase in ROS generation; however, it is still unknown whether AD is primary or secondary to excess oxidative stress. As Aβ can easily penetrate the BBB and is generally acknowledged as the oldest AD species, it is a promising choice as a blood biomarker. However, it has not been approved as a reliable signal for blood analysis, likely due to contradictory research findings. One study revealed [33] that the Aβ deposited in the brain is composed of 42 amino acids (Aβ-42) [37]. Many variants of Aβ are present in patients with AD, but the levels of Aβ-40 and Aβ-42 in CSF are the most reliable indicators of the disease. Although this amyloidogenic protein is present in the human body, it has been observed more than once that Aβ-42 concentrations in the CSF of patients correlate with Aβ levels in the brain [38]. Aβ-40, the most prevalent isoform of Aβ that is found in CSF, is another derivative of Aβ, which may function as a possible biomarker for AD. Many studies looking at the levels of A in those at risk for AD have found that Aβ-40 or Aβ-42 [39] levels are raised. In addition, Aβ-40 or Aβ-42 concentrations are decreased in people who are at risk for AD, or Aβ-40, and Aβ-42 levels have no bearing on a patient’s likelihood of developing dementia [40,41]. Our research demonstrates that H_2_ gas inhalation for four weeks following therapy decreased the level of the Aβ-40/Aβ-42 ratio in post-treatment groups compared to pre-treatment groups and improved cognitive impairments in AD patients, which is shown in Figure 5 and Figure S3.

High levels of CSF t-tau have also been described, in earlier studies, as a sign of more serious cognitive impairment [42], fast progression to AD [43], and conversion to moderate dementia [44]. Based on these findings, it can be concluded that significantly [45] elevated CSF t-tau levels, indicating greater neuronal loss, are associated with a higher propensity for extensive cortical changes. The negative correlation between CSF p-tau levels and neuronal loss outside the medial temporal areas in individuals with high tau levels suggests the possibility of more extensive neuronal loss [46]. Our results suggested that t-Tau and p-Tau levels were significantly reduced after 4 weeks of H_2_ gas inhalation treatment, which was effective for improved neurodegenerative diseases, such as AD, which is shown in Figure 5 and Figure S4.

Immune dysregulation promotes neurodegeneration and is a role in cognitive decline, including synapse loss and neuronal death. It is characterized by persistent and increasing glial polarization [47]. Age-related cognitive decline is associated with plasma MCP-1 levels and functional and anatomical changes in the brain [48]. This result supports the newly developed theory that intrathecal inflammation occurs before the clinical onset of AD [48]. The fact that MCP-1 levels are elevated in the serum of patients with MCI and mild-to-moderate AD, whereas they decline throughout AD development, lends credence to this finding. Our results suggest that H_2_ gas inhalation improves cognitive function in patients with dementia by decreasing MCP-1 levels, indicating that H_2_ might be a good candidate for improving AD with cognitive dysfunction, as shown in Figure 6 and Figure S5. It was reported that H_2_-rich water improved cognitive impairment [49]. Clinical testing revealed that 72 h of exposure to 2.4% H_2_ gas had no negative impacts on any physiological measures, indicating that H_2_ may not have any negative effects [50].

VEGF-A is a risk factor for chronic diseases, including AD. Although, the VEGF family is crucial for regulating angiogenic activity, neurogenesis, and neuronal survival [48]. Although the results have not been completely consistent, VEGF has been investigated as a possible biomarker of AD. In one study, the intrathecal levels of VEGF in the CSF of individuals with AD and vascular dementia were higher than those in healthy controls (i.e., no neurological disease or deficit) [51]. Another study discovered that there was no difference in CSF VEGF levels between patients with AD and cognitively healthy controls [52]. Similarly, VEGF promoter polymorphisms increased the risk of AD, although there was no change in serum VEGF levels between patients with AD and control [53]. Additionally, the build-up of VEGF with Aβ plaques in the brains of patients with AD may sequester VEGF, leading to a shortage [54]. Our findings leave us uncertain of whether the correlation between low blood VEGF levels and AD is due to the primary protective role of VEGF against AD or a secondary effect of AD pathology, such as Aβ plaques, on serum VEGF levels, which is shown in Figure 6 and Figure S5. Our results also reveal that H_2_ gas inhalation significantly decreased dementia biomarkers by reducing oxidative stress and enhancing antioxidant activity.

## 5. Conclusions

In summary, H_2_ gas inhalation in the elderly is a good candidate for improving the risk of developing a neurodegenerative disease (dementia). However, further mechanistic studies are needed to fully elucidate the effects of H_2_ gas inhalation in community-dwelling adults of different ages.

## Figures and Tables

**Table 1:** Inclusion and exclusion criteria.

Inclusion Criteria	Exclusion Criteria
Patients aged 40 to 70 years can walk voluntarily.	Those who have a history of AD disease.
Patients who are willing or able to follow the doctor’s instructions, including joint movements.	Those who have participated in other clinical trials within 6 months of participating in clinical trials.
Persons who can comply with concomitantly permitted drugs, prohibited drugs, and relief drugs.	Patients who have another autoimmune disease.
Persons who can maintain the same exercise and activity during the clinical trial period.	Patients with tumors other than neurodegenerative diseases.
Fully understand the purpose and procedure of this clinical trial.	Pregnant and lactating women.

**Table 2:** Baseline characteristics of participants.

Variable		Total	Female	Male
Gender		51	39 (76.47%)	11 (21.57%)
Age (years)		53.98 ± 8.86	56.15 ± 8.89	46.09 ± 7.91
Education		51	Graduation	Graduation
Blood Pressure (mmHg)	Systolic	123.43 ± 8.44	122.82 ± 7.80	125.63 ± 6.93
	Diastolic	78.07 ± 7.60	77.67 ± 7.79	79.54 ± 4.20

## Data Availability

The data are contained within the article and supplementary materials.

## Supplementary Materials

The following supporting information can be downloaded at: https://www.mdpi.com/article/10.3390/antiox12061241/s1, Figure S1: Effects of H_2_ gas inhalation on serum BACE-1 levels before and after treatment in different ages (50–59 years, 60–69 years, and 70–79 years); Figure S2: Effects of H_2_ gas inhalation on serum BDNF levels before and after treatment in different ages (50–59 years, 60–69 years, and 70–79 years); Figure S3: Effects of H_2_ gas inhalation on serum Aβ-40 and Aβ-42 levels before and after treatment in different ages (50–59 years, 60–69 years, and 70–79 years); Figure S4: Effects of H_2_ gas inhalation on serum t-Tau and p-Tau levels before and after treatment in different ages (50–59 years, 60–69 years, and 70–79 years); Figure S5: Effects of H_2_ gas inhalation on serum MCP-1 and VEGF-A levels before and after treatment in different ages (50–59 years, 60–69 years, and 70–79 years).
