# Supplementary material for: Effects of Hydrogen Gas Inhalation on Community-Dwelling Adults of Various Ages: A Single-Arm, Open-Label, Prospective Clinical Trial
Source: Antioxidants (Basel). 2023 Jun 8;12(6):1241. doi: 10.3390/antiox12061241 (PMC10295751; doi:10.3390/antiox12061241)
Supplement: Supplementary file 1 [file antioxidants-12-01241-s001.zip › antioxidants-2372945-supplementary.pdf]

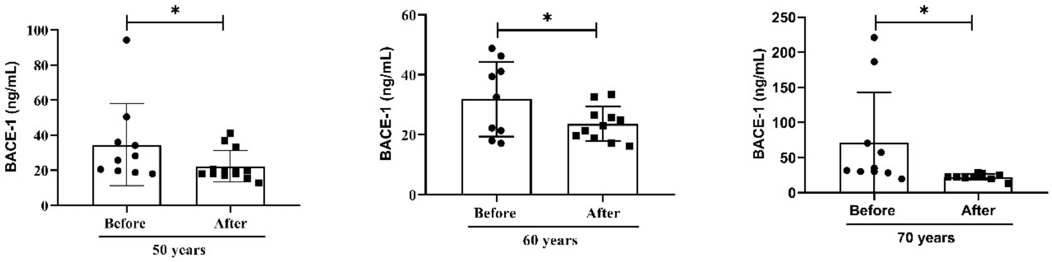

Figure S1

Figure S1. Effects of H_2 gas inhalation on serum BACE-1 levels before and after treatment in different ages (50–59 years, 60-69 years, and 70–79 years). Data are presented as mean ± SD.* $p < 0.05$.

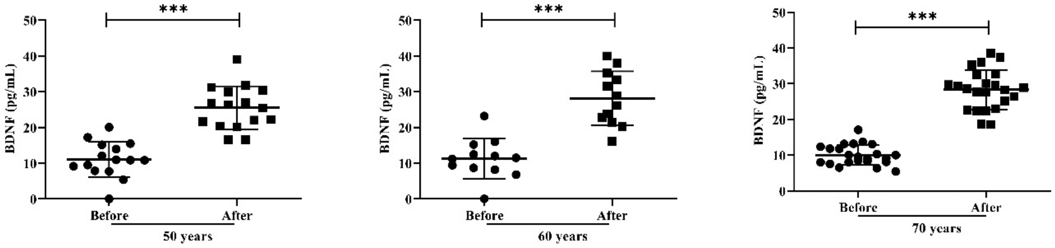

Figure S2. Effects of H_2 gas inhalation on serum BDNF levels before and after treatment in different ages (50–59 years, 60–69 years, and 70–79 years). Data are presented as mean ± SD.*** $p < 0.001$.

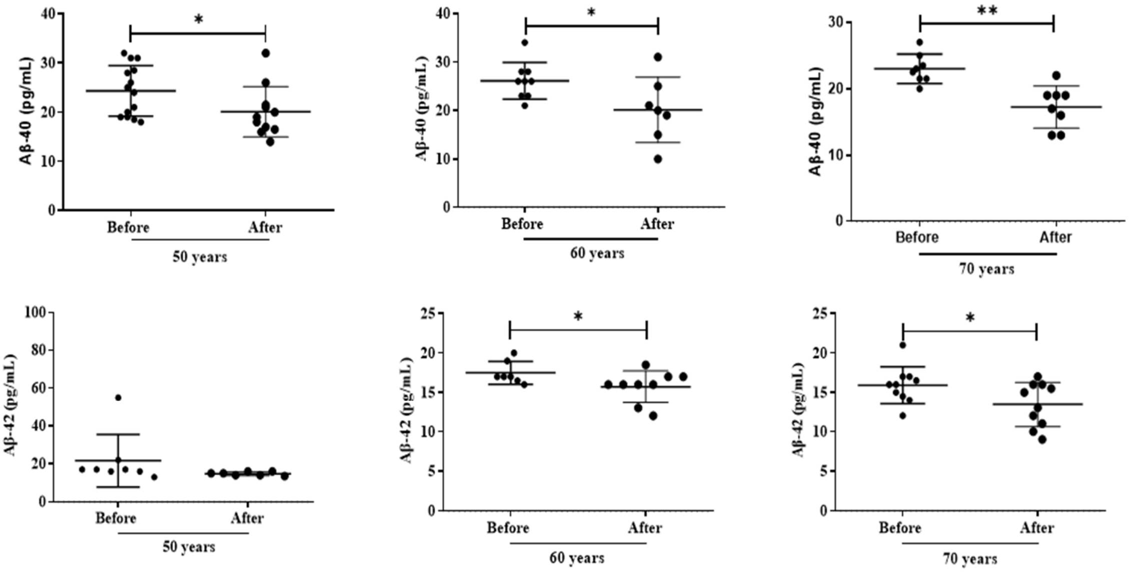

Figure S3. Effects of H_2 gas inhalation on serum Aβ-40 and Aβ-42 levels before and after treatment in different ages (50–59 years, 60–69 years, and 70–79 years). Data are presented as mean ± SD.** $p < 0.01$, * $p < 0.05$.

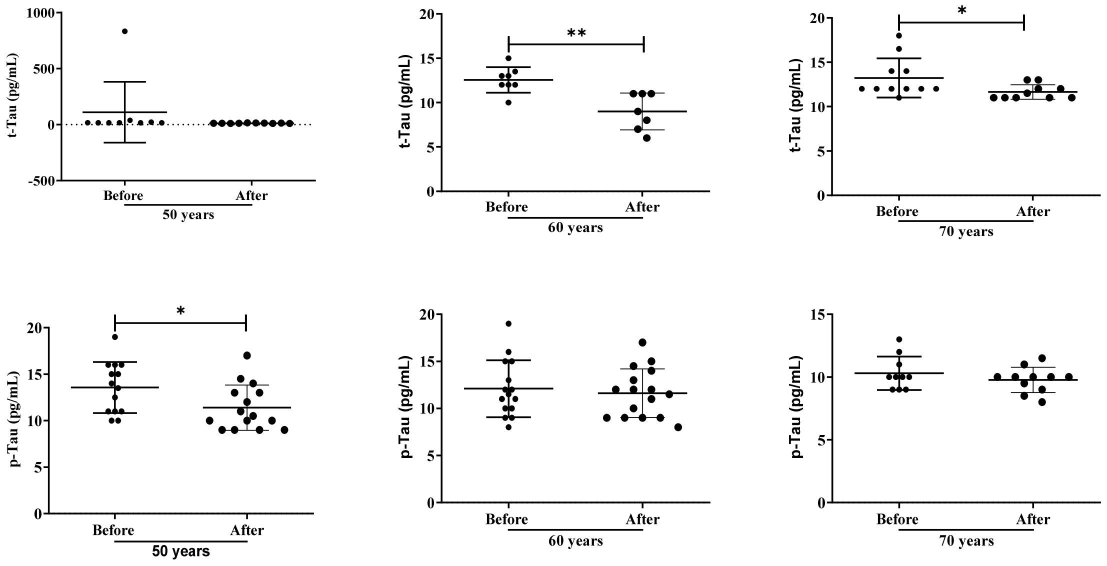

Figure S4. Effects of H_2 gas inhalation on serum t-Tau and p-Tau levels before and after treatment in different ages (50–59 years,60–69 years, and 70–79 years). Data are presented as mean ± SD.** $p < 0.01$, * $p < 0.05$.

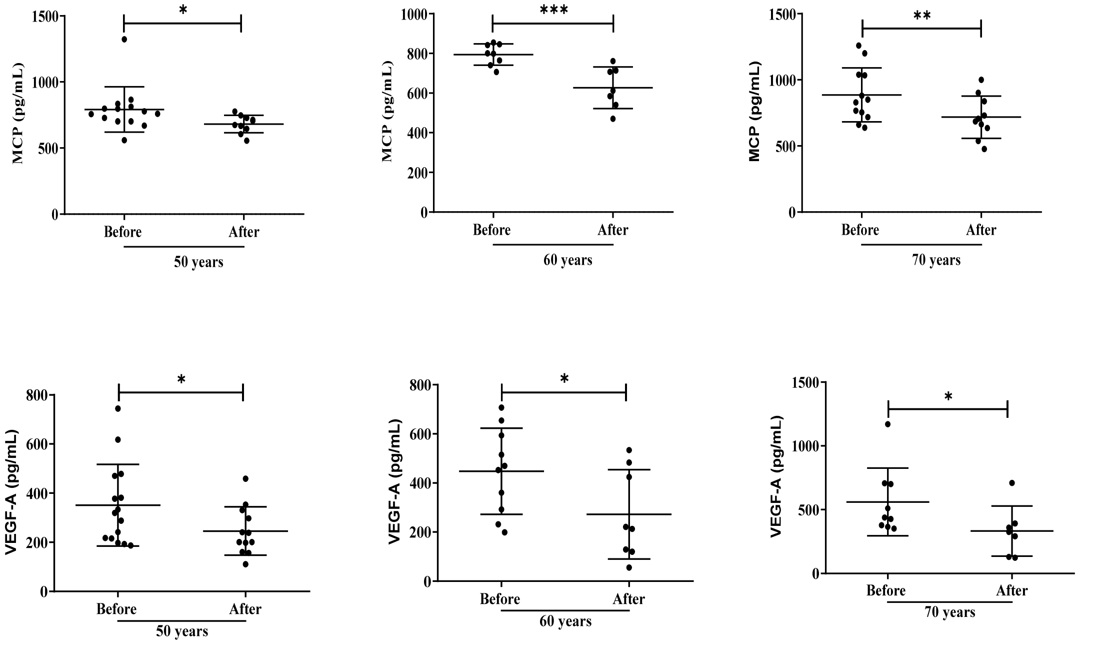

Figure S5. Effects of H_2 gas inhalation on serum MCP-1 and VEGF-A levels before and after treatment in different ages (50–59 years, 60–69 years, and 70–79 years). Data are presented as mean ± SD. *** $p < 0.001$, ** $p < 0.01$, * $p < 0.05$.
